# Supplementary figures and images for: Rudhira-mediated microtubule stability controls TGFβ signaling during mouse vascular development
Source: eLife. 2025 May 15;13:RP98257. doi: 10.7554/eLife.98257 (PMC12080998; doi:10.7554/eLife.98257)

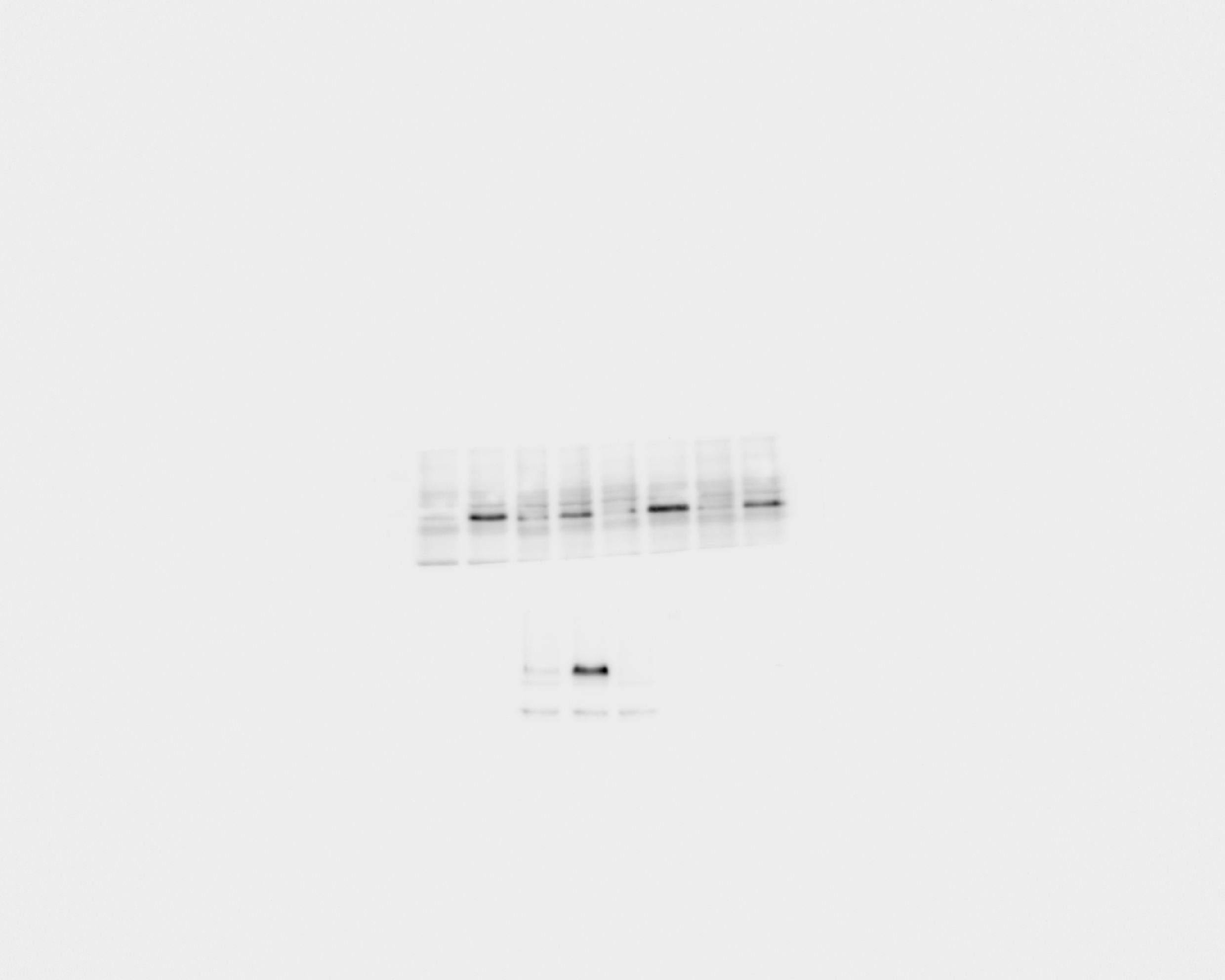

Supplement: Figure 1—source data 2. [file elife-98257-fig1-data2.zip › Fig1_SourceData1_raw/Fig1C_pSMAD23_raw.jpg]

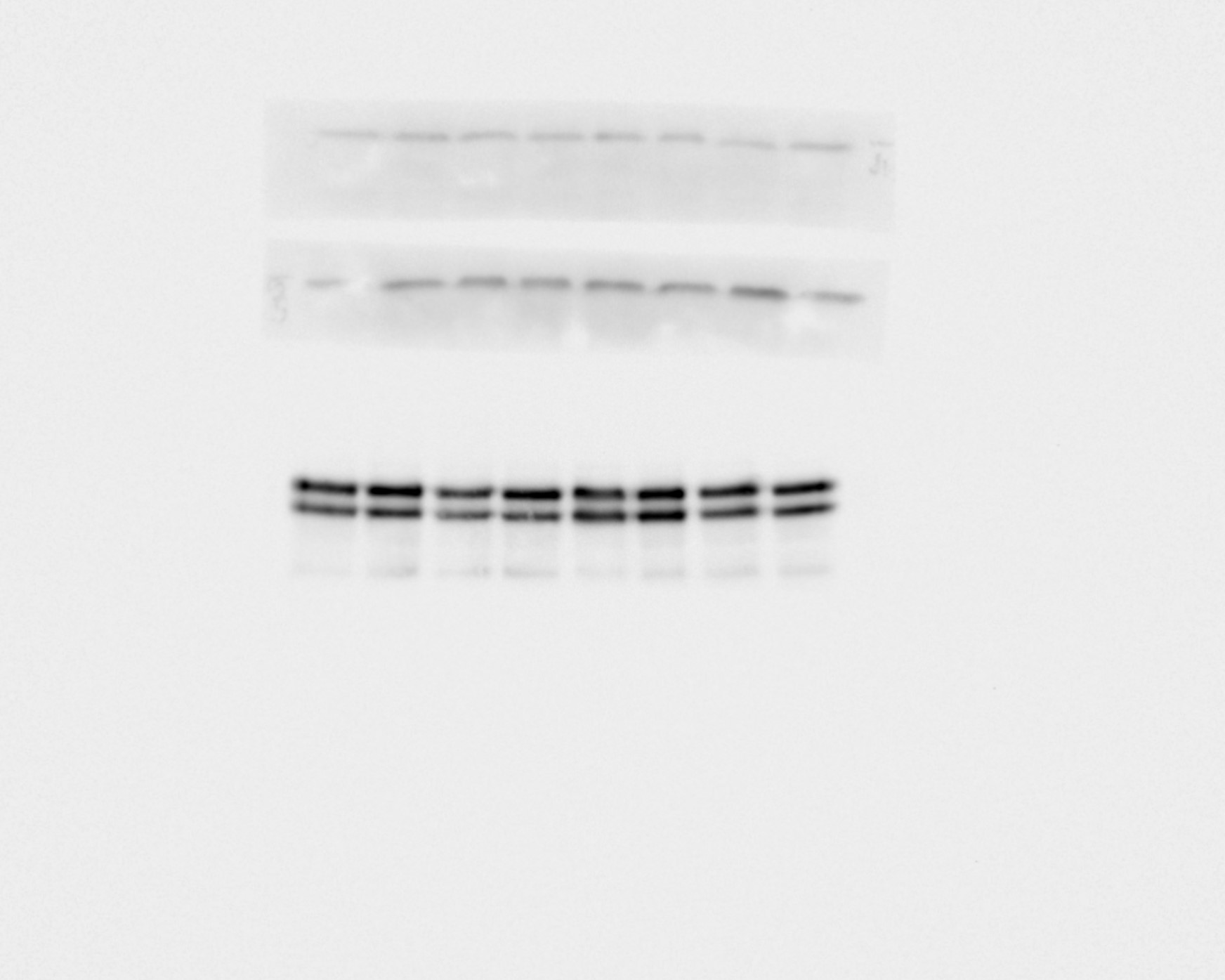

Supplement: Figure 1—source data 2. [file elife-98257-fig1-data2.zip › Fig1_SourceData1_raw/Fig1C_SMAD23_raw.jpg]

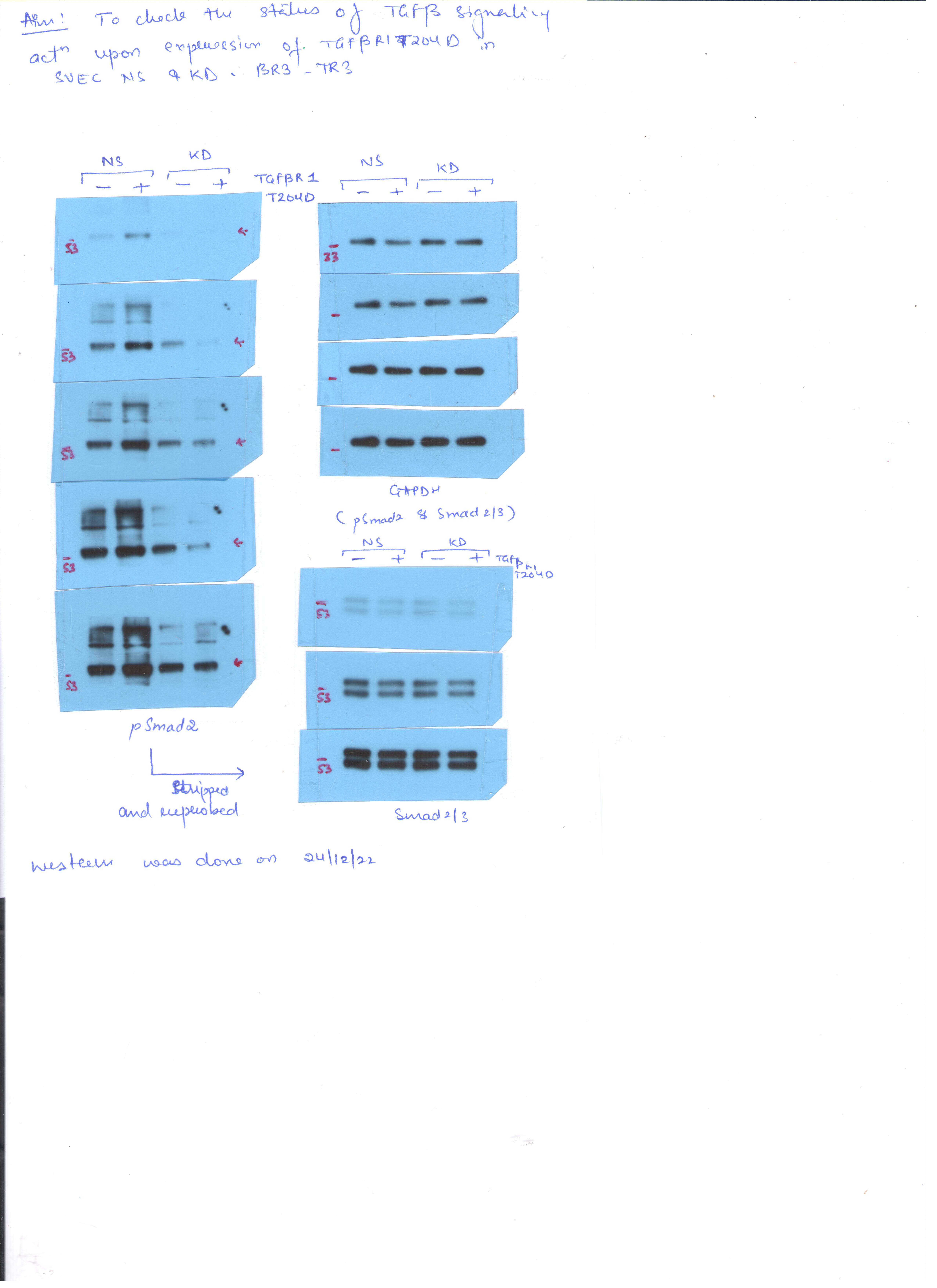

Supplement: Figure 1—source data 2. [file elife-98257-fig1-data2.zip › Fig1_SourceData1_raw/Fig1B_raw.jpg]

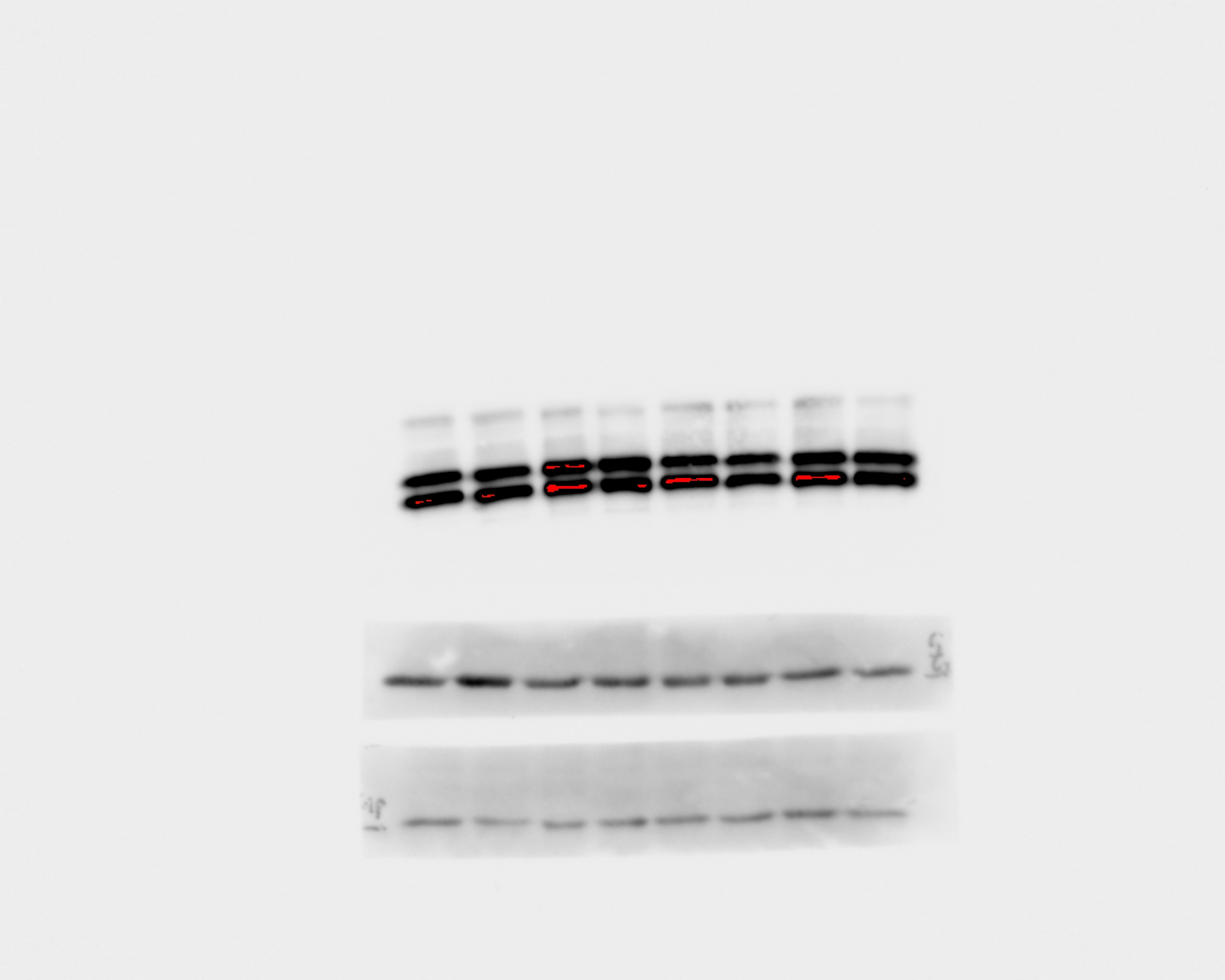

Supplement: Figure 1—source data 2. [file elife-98257-fig1-data2.zip › Fig1_SourceData1_raw/Fig1C GAPDH_raw.jpg]

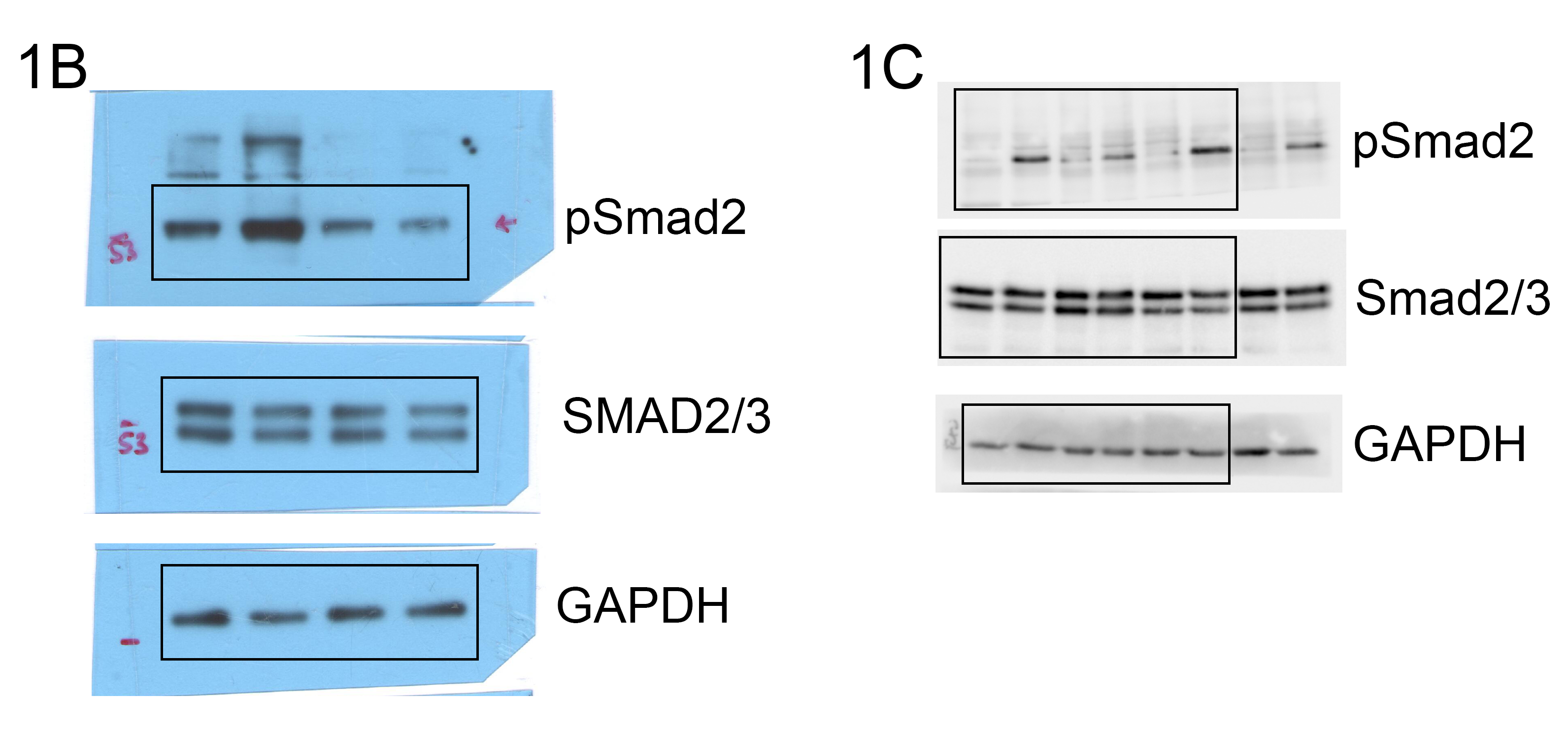

Supplement: Figure 1—source data 3. [file elife-98257-fig1-data3.zip › Fig1_SourceData2/Fig1_SourceData2.tif]

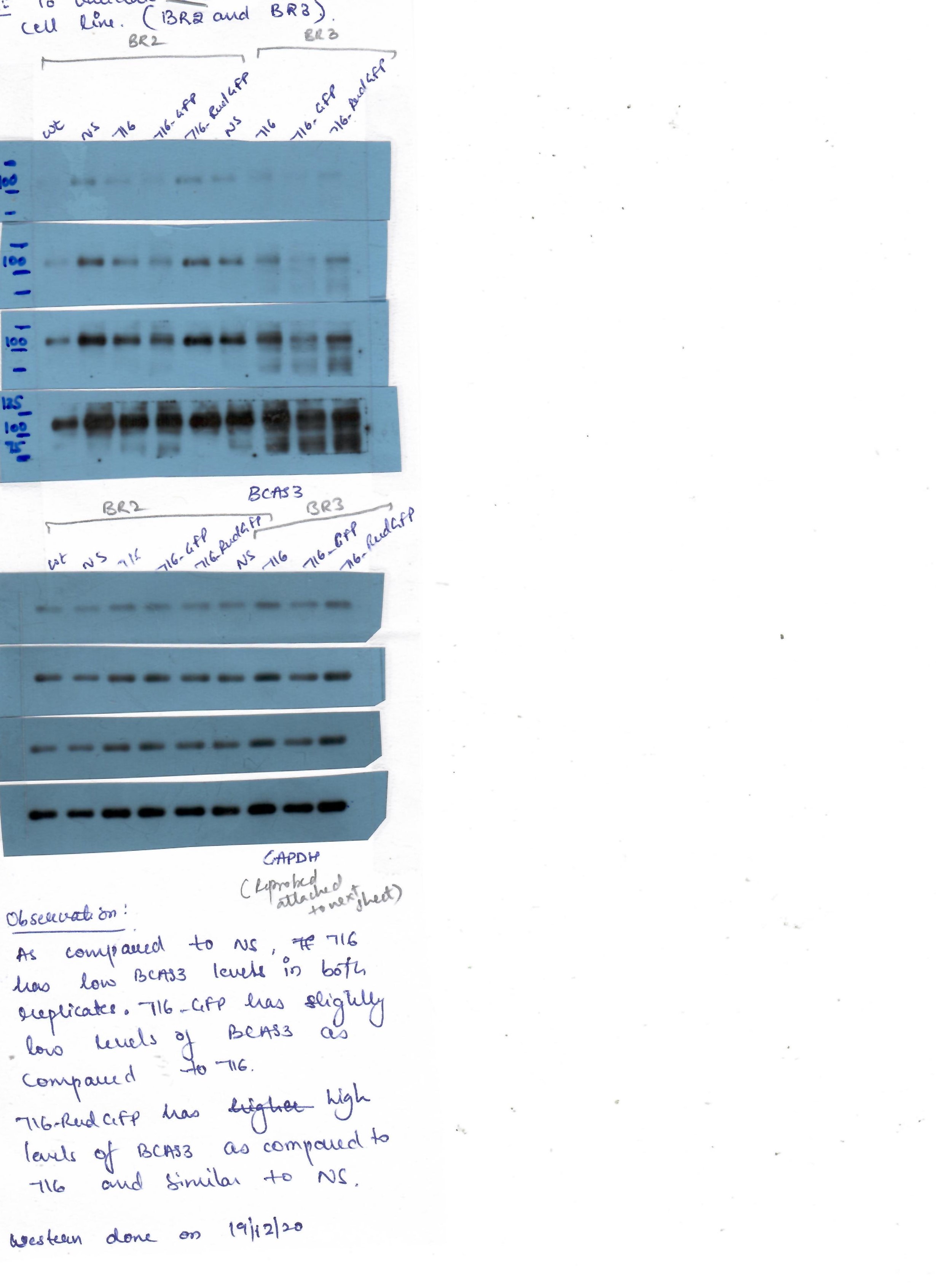

Supplement: Figure 1—figure supplement 1—source data 2. [file elife-98257-fig1-figsupp1-data2.zip › Fig1-Fig Supple1_SourceData1_raw/Fig1- Fig Supple1B_raw.jpeg]

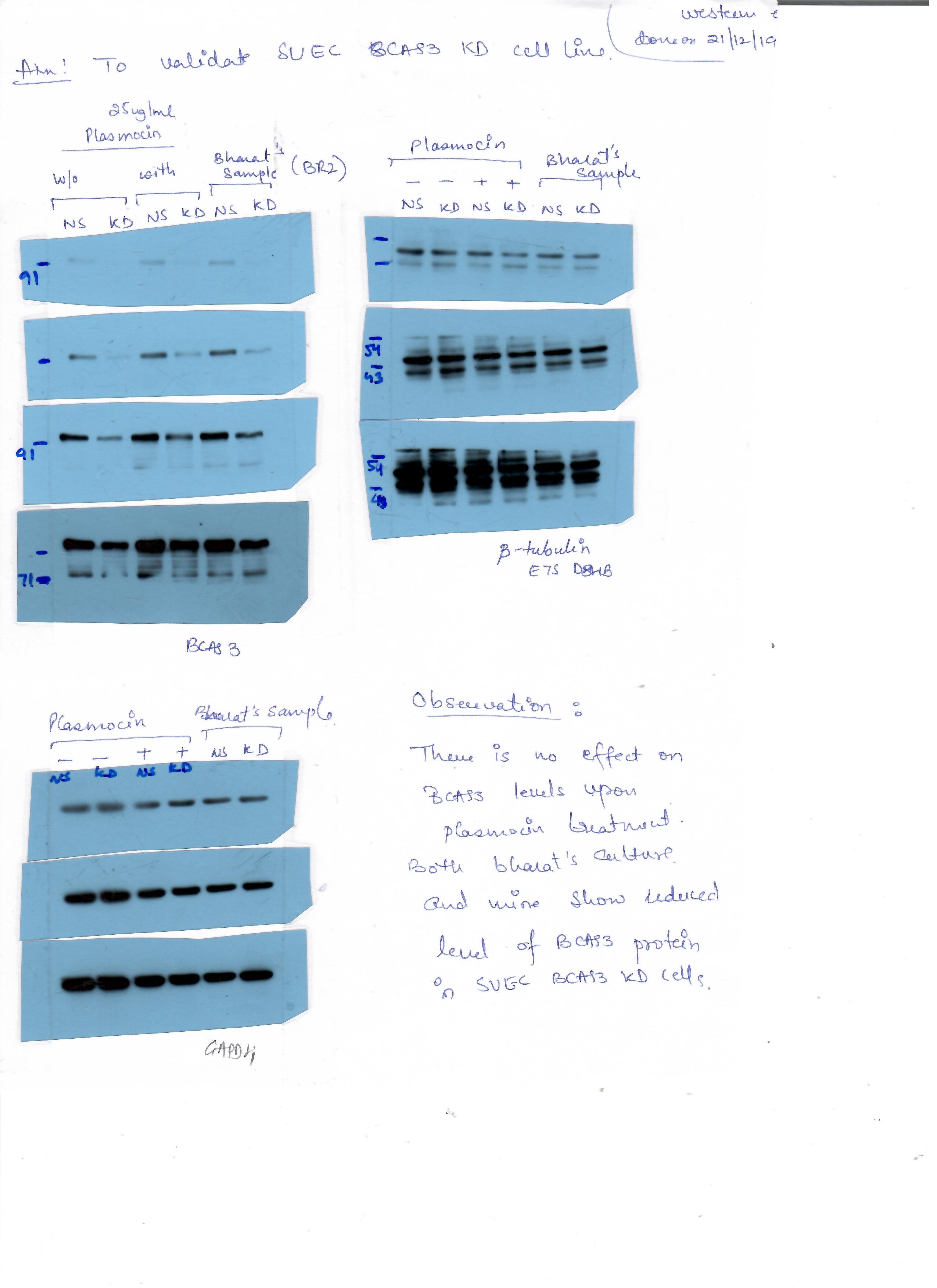

Supplement: Figure 1—figure supplement 1—source data 2. [file elife-98257-fig1-figsupp1-data2.zip › Fig1-Fig Supple1_SourceData1_raw/Fig1- Fig Supple1A_raw.jpeg]

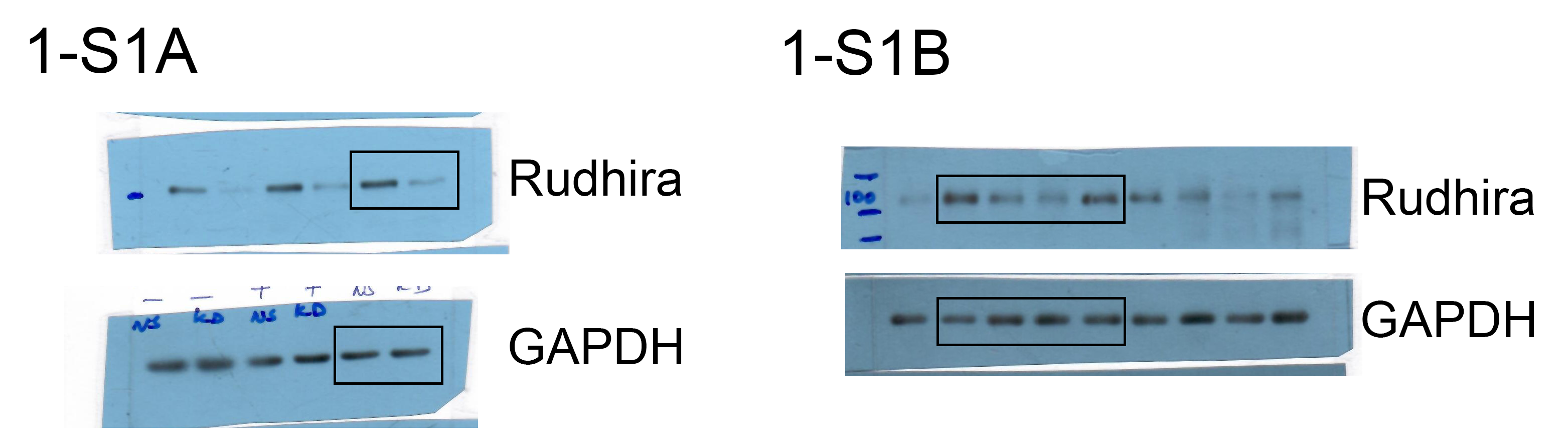

Supplement: Figure 1—figure supplement 1—source data 3. [file elife-98257-fig1-figsupp1-data3.zip › Fig1-Fig Supple1_SourceData2/Fig1-Fig Supple1_SourceData2.tif]

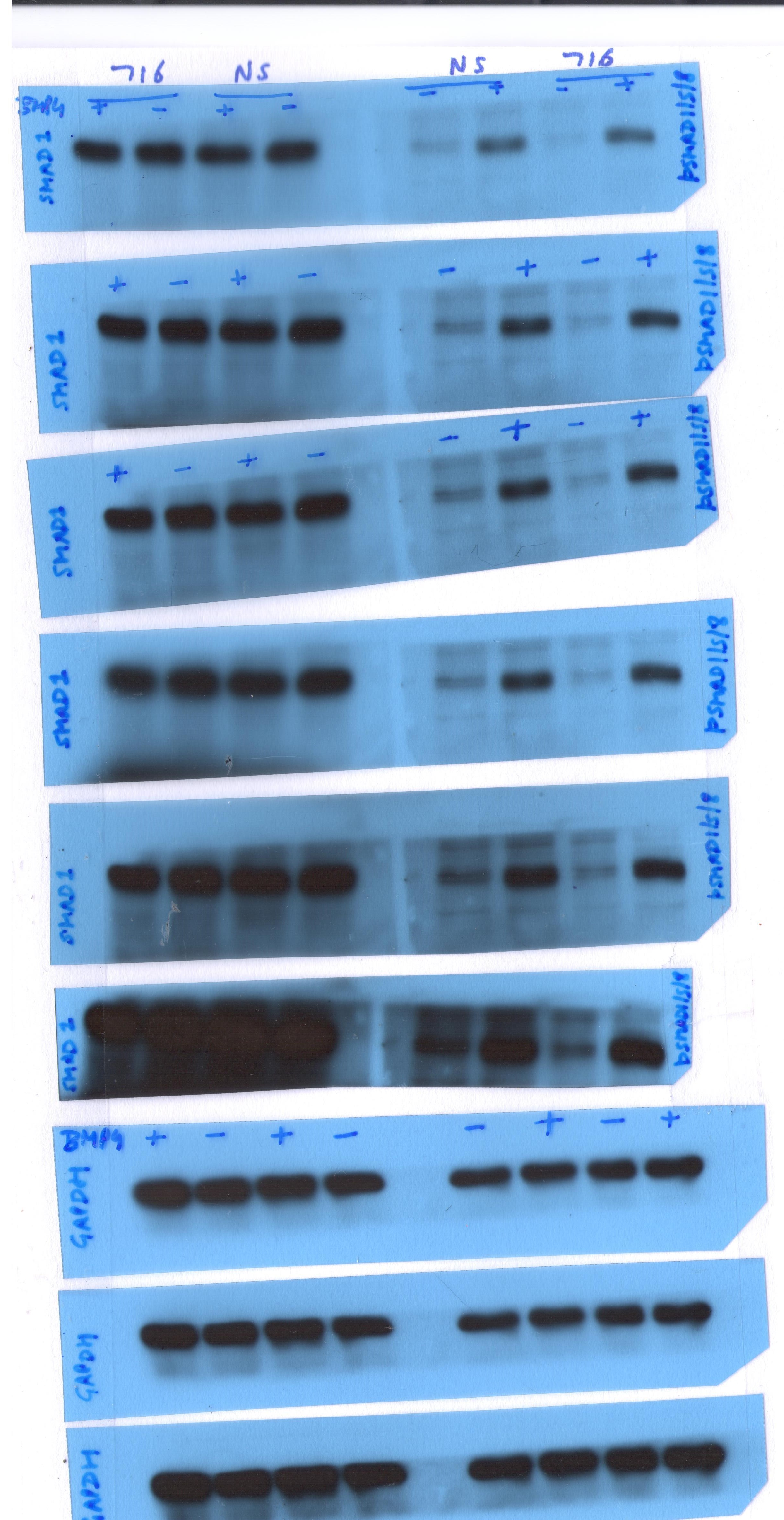

Supplement: Figure 1—figure supplement 2—source data 2. [file elife-98257-fig1-figsupp2-data2.zip › Fig1-Fig Supple2_SourceData1_raw/Fig1- Fig Supple2D.jpg]

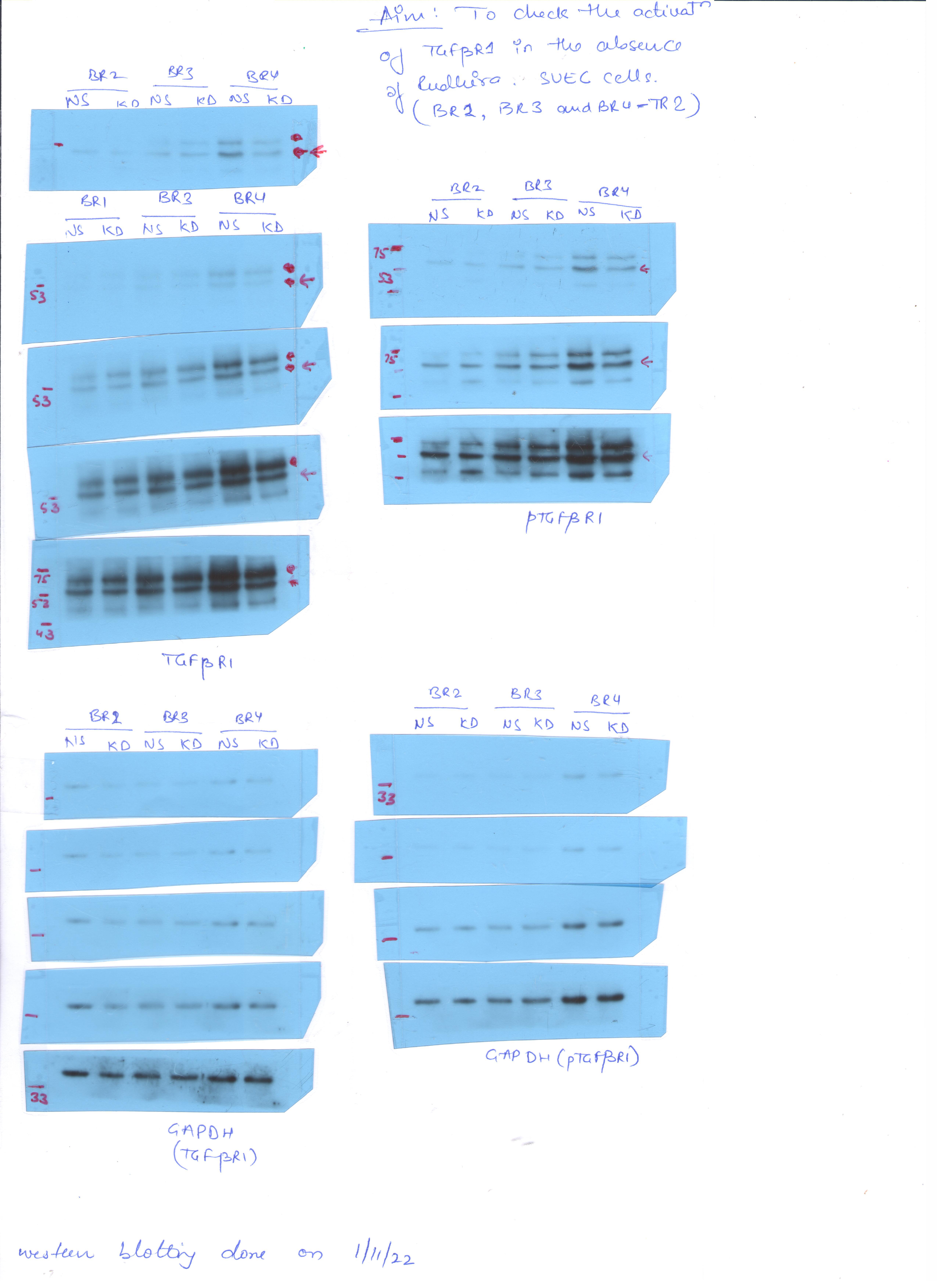

Supplement: Figure 1—figure supplement 2—source data 2. [file elife-98257-fig1-figsupp2-data2.zip › Fig1-Fig Supple2_SourceData1_raw/Fig1- Fig Supple2B_raw.jpg]

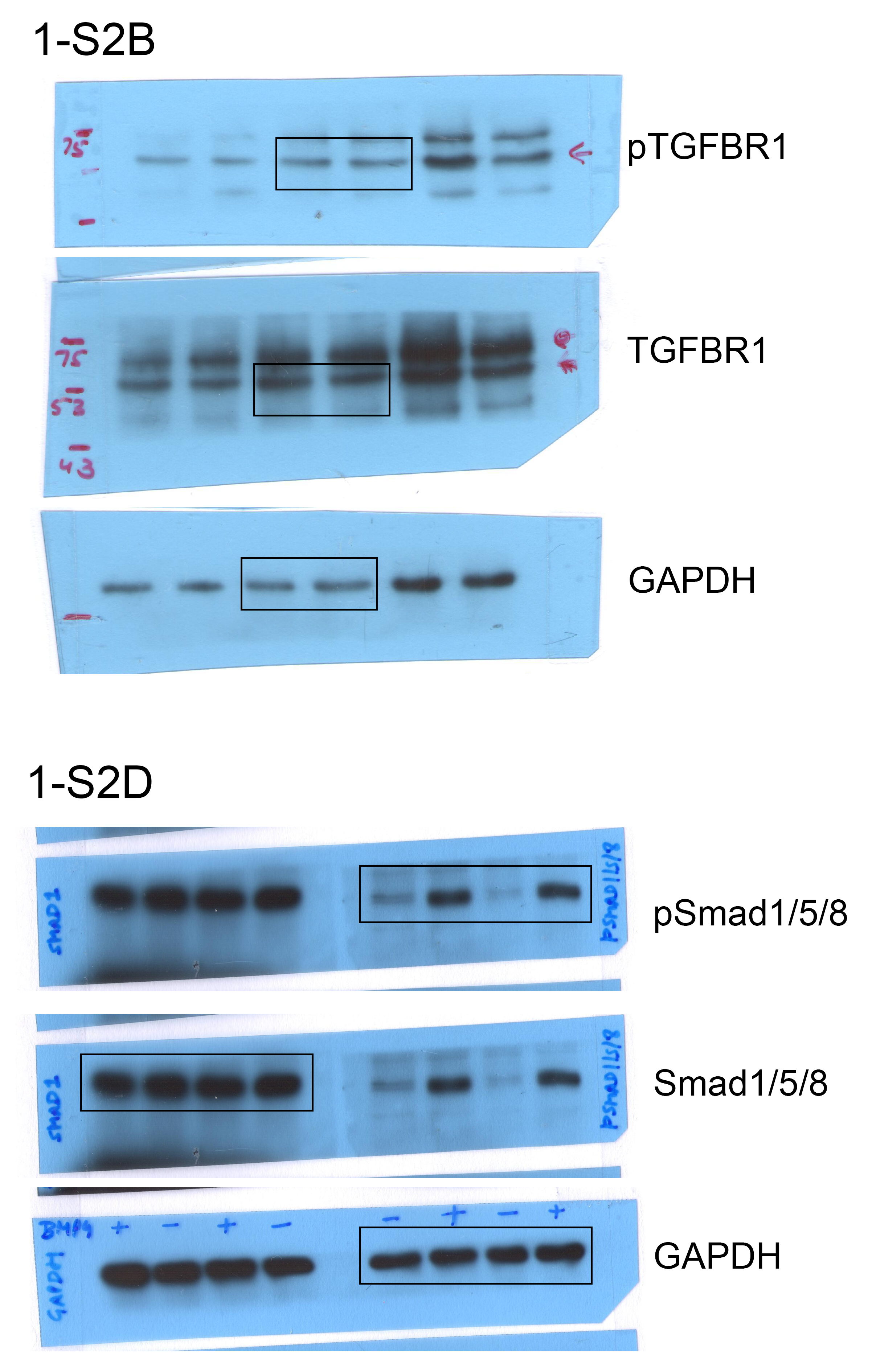

Supplement: Figure 1—figure supplement 2—source data 3. [file elife-98257-fig1-figsupp2-data3.zip › Fig1-Fig Supple2_SourceData2/Fig1-Fig Supple2_SourceData2.tif]

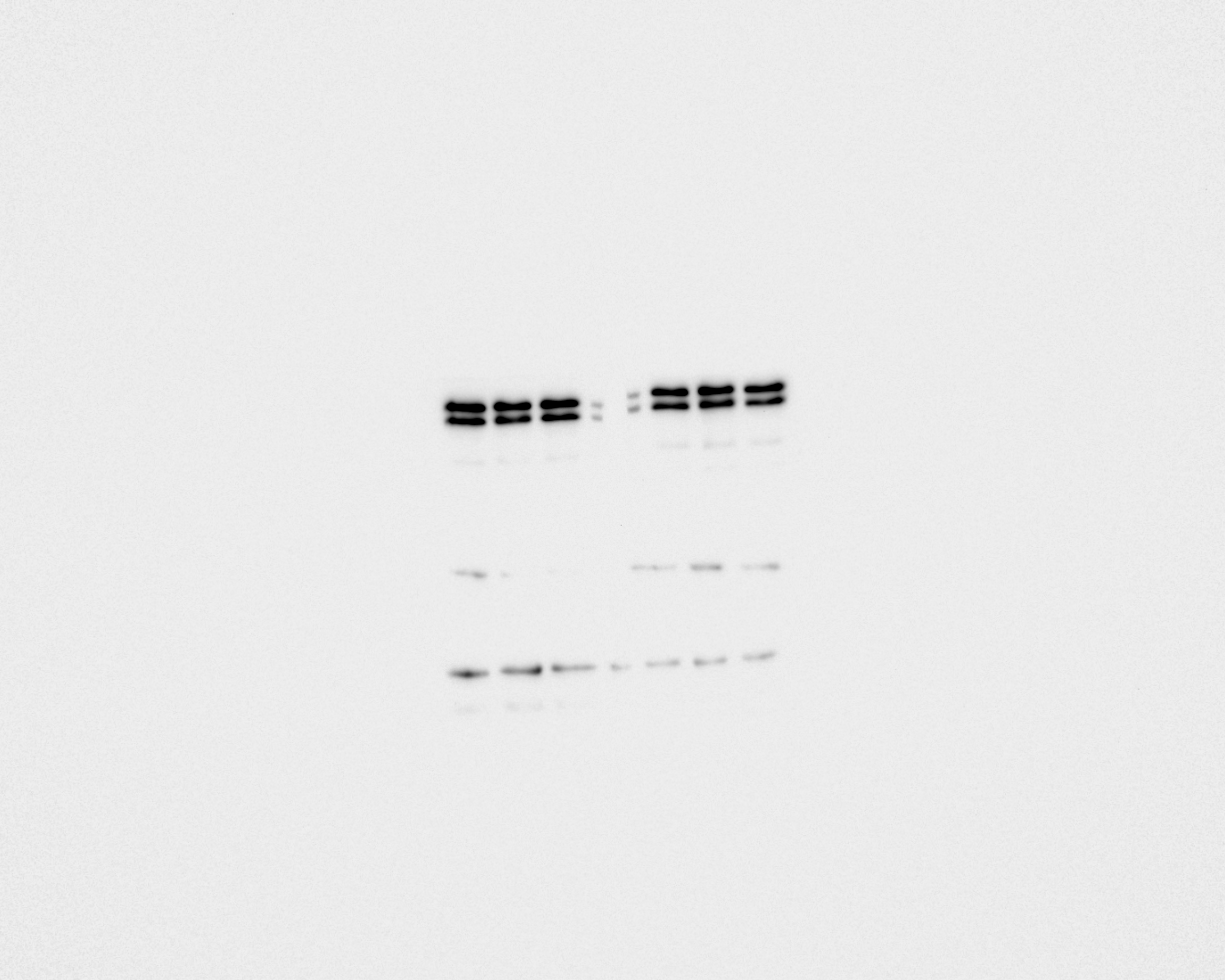

Supplement: Figure 1—figure supplement 3—source data 2. [file elife-98257-fig1-figsupp3-data2.zip › Fig1-Fig Supple3_SourceData1_raw/Fig1- Fig Supple3A_Smad23, GAPDH.tif]

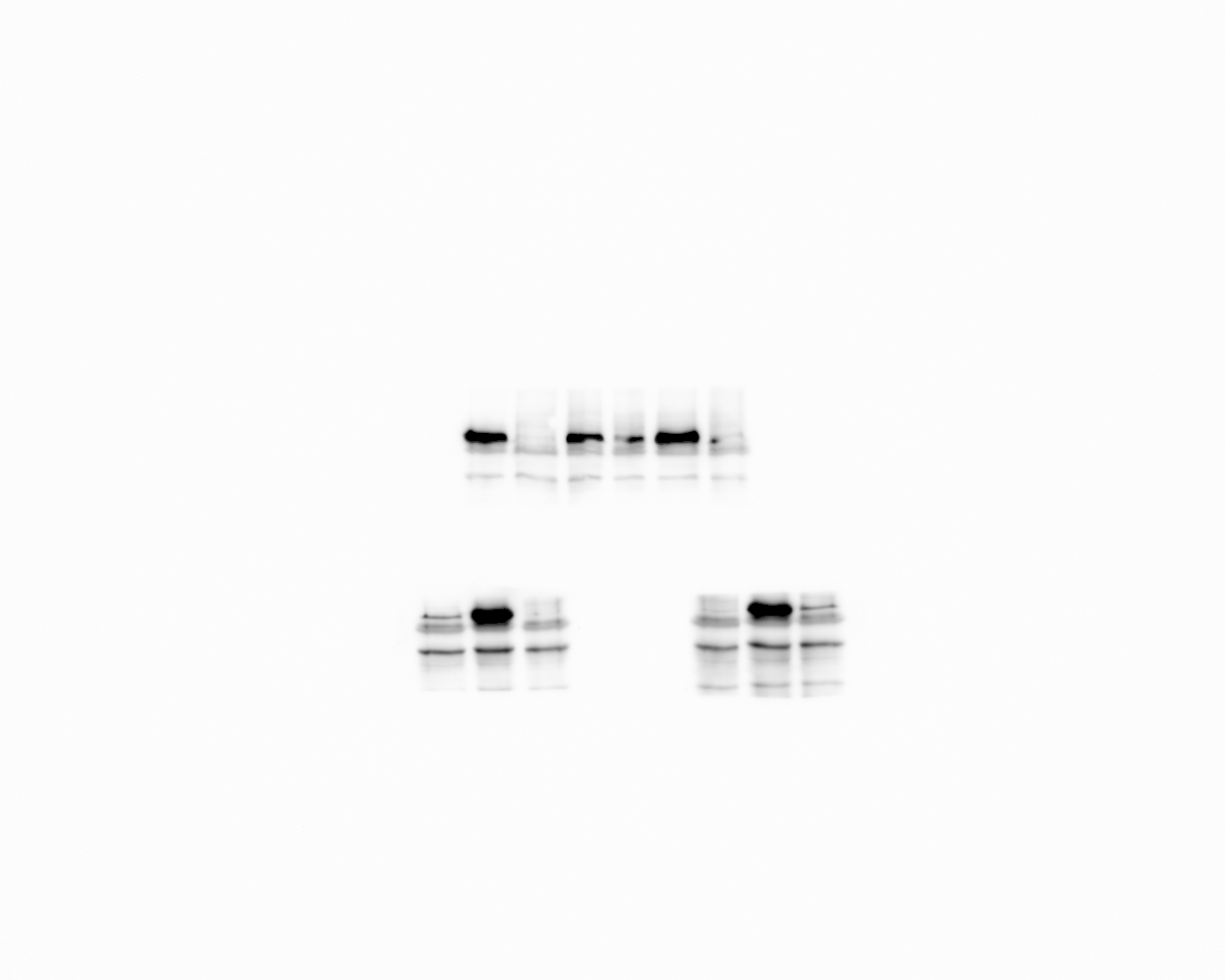

Supplement: Figure 1—figure supplement 3—source data 2. [file elife-98257-fig1-figsupp3-data2.zip › Fig1-Fig Supple3_SourceData1_raw/Fig1- Fig Supple3A_pSmad2.tif]

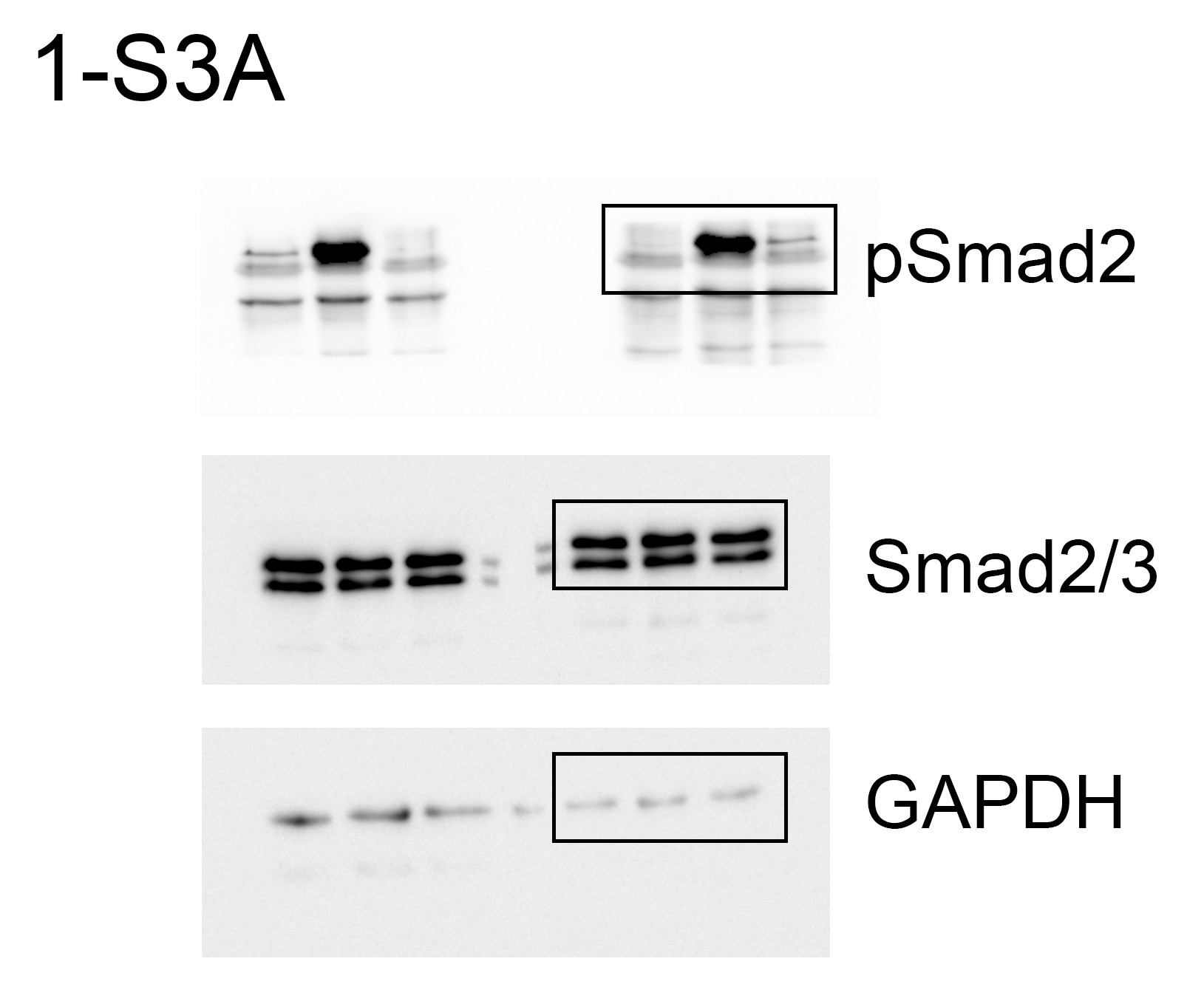

Supplement: Figure 1—figure supplement 3—source data 3. [file elife-98257-fig1-figsupp3-data3.zip › Fig1-Fig Supple3_SourceData2/Fig1-Fig Supple3_SourceData2.tif]

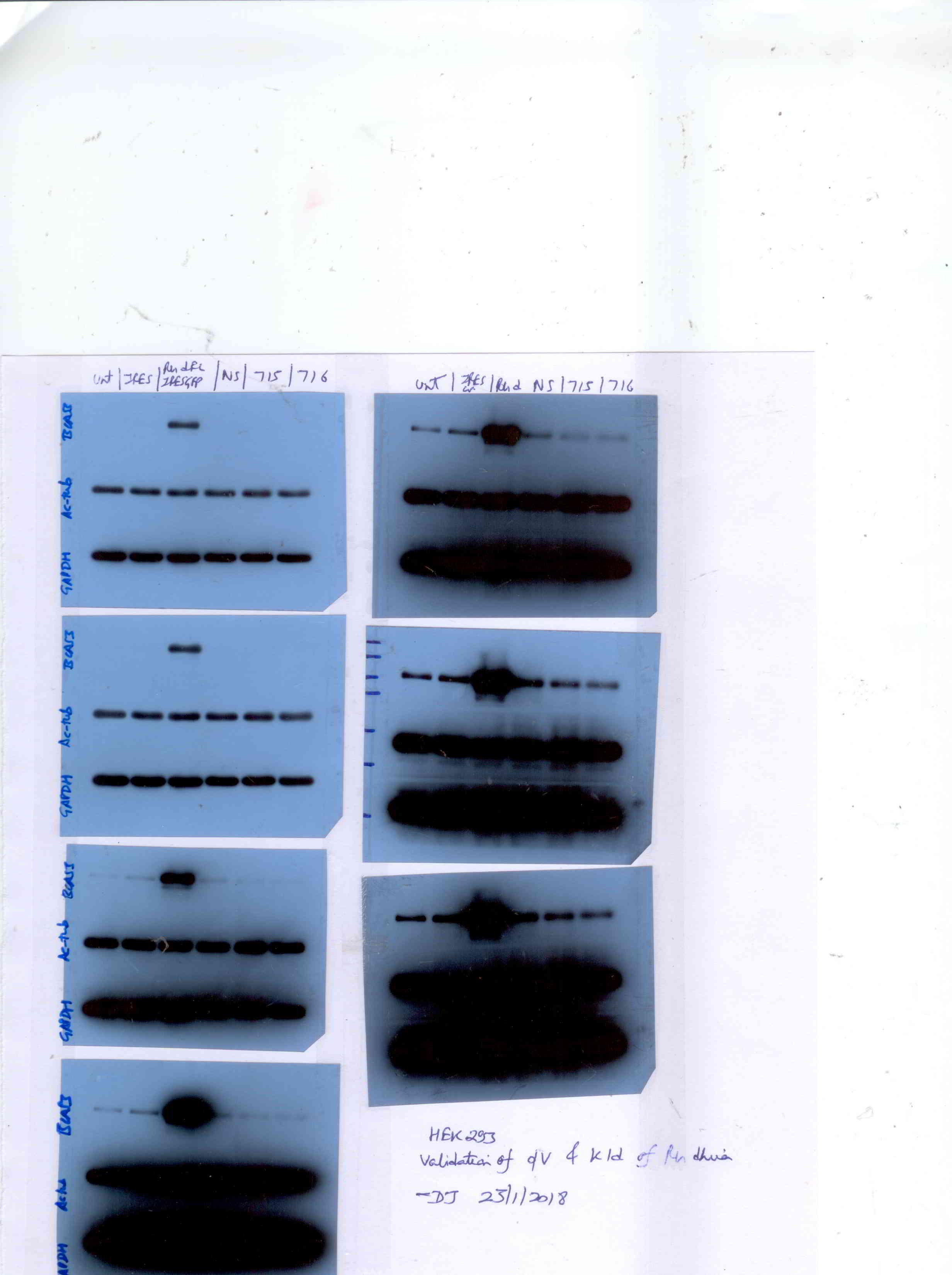

Supplement: Figure 2—source data 2. [file elife-98257-fig2-data2.zip › Fig2_SourceData1_raw/Fig2C_raw.jpeg]

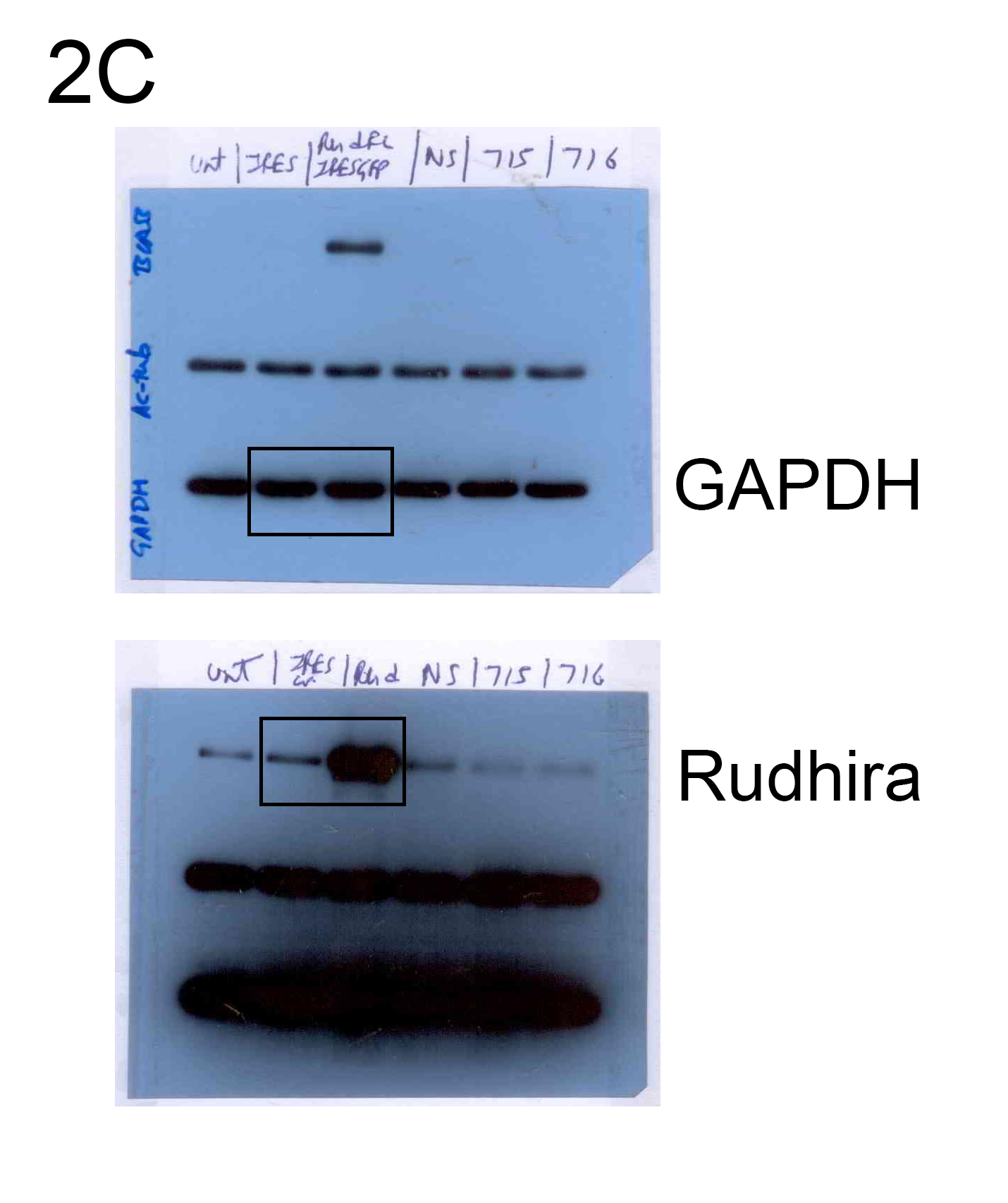

Supplement: Figure 2—source data 3. [file elife-98257-fig2-data3.zip › Fig2_SourceData2/Fig2_SourceData2.tif]

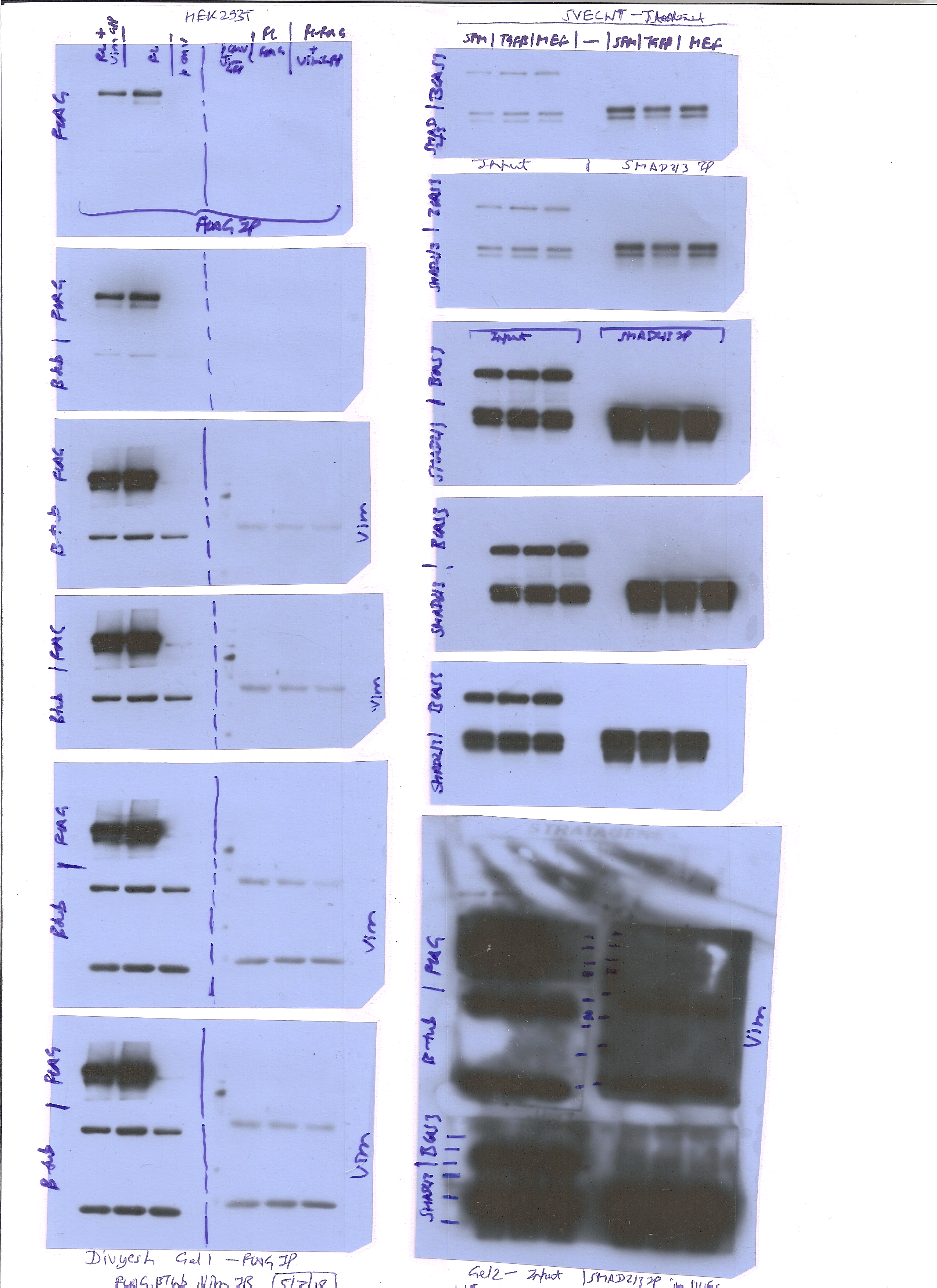

Supplement: Figure 3—source data 2. [file elife-98257-fig3-data2.zip › Fig3_SourceData1_raw/Fig3A_raw.tiff]

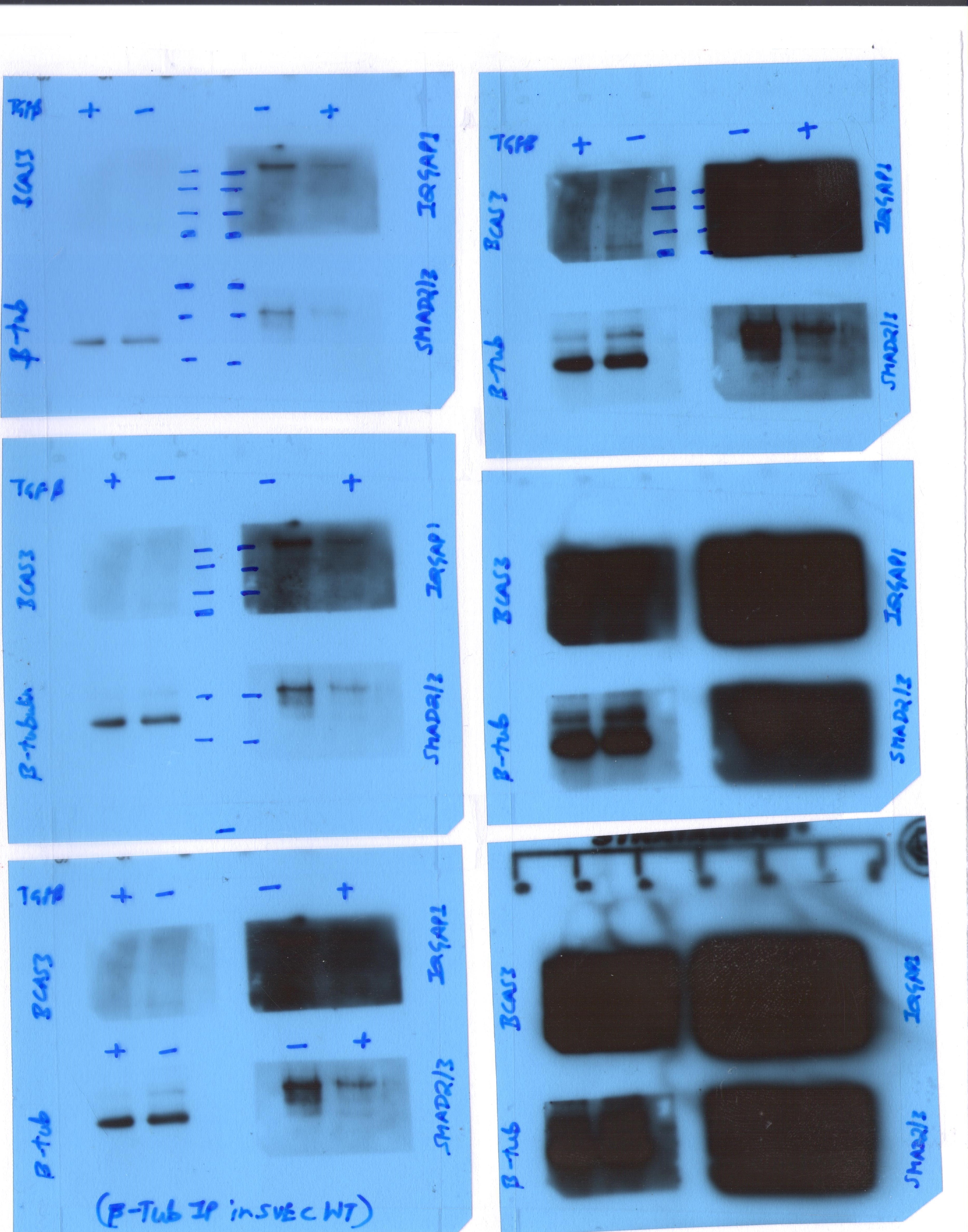

Supplement: Figure 3—source data 2. [file elife-98257-fig3-data2.zip › Fig3_SourceData1_raw/Fig3B_raw.jpg]

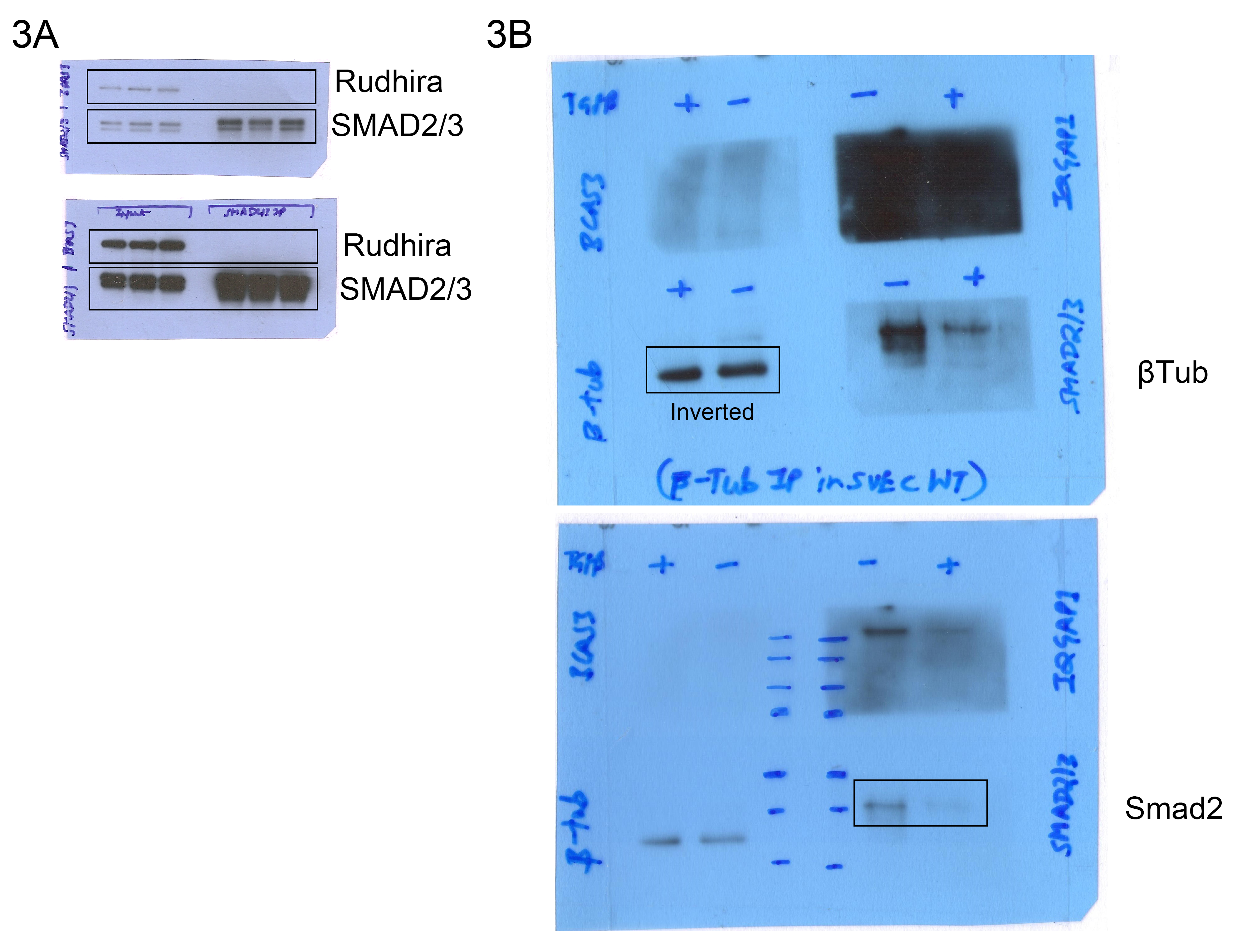

Supplement: Figure 3—source data 3. [file elife-98257-fig3-data3.zip › Fig3_SourceData2/Fig3_SourceData2.tif]

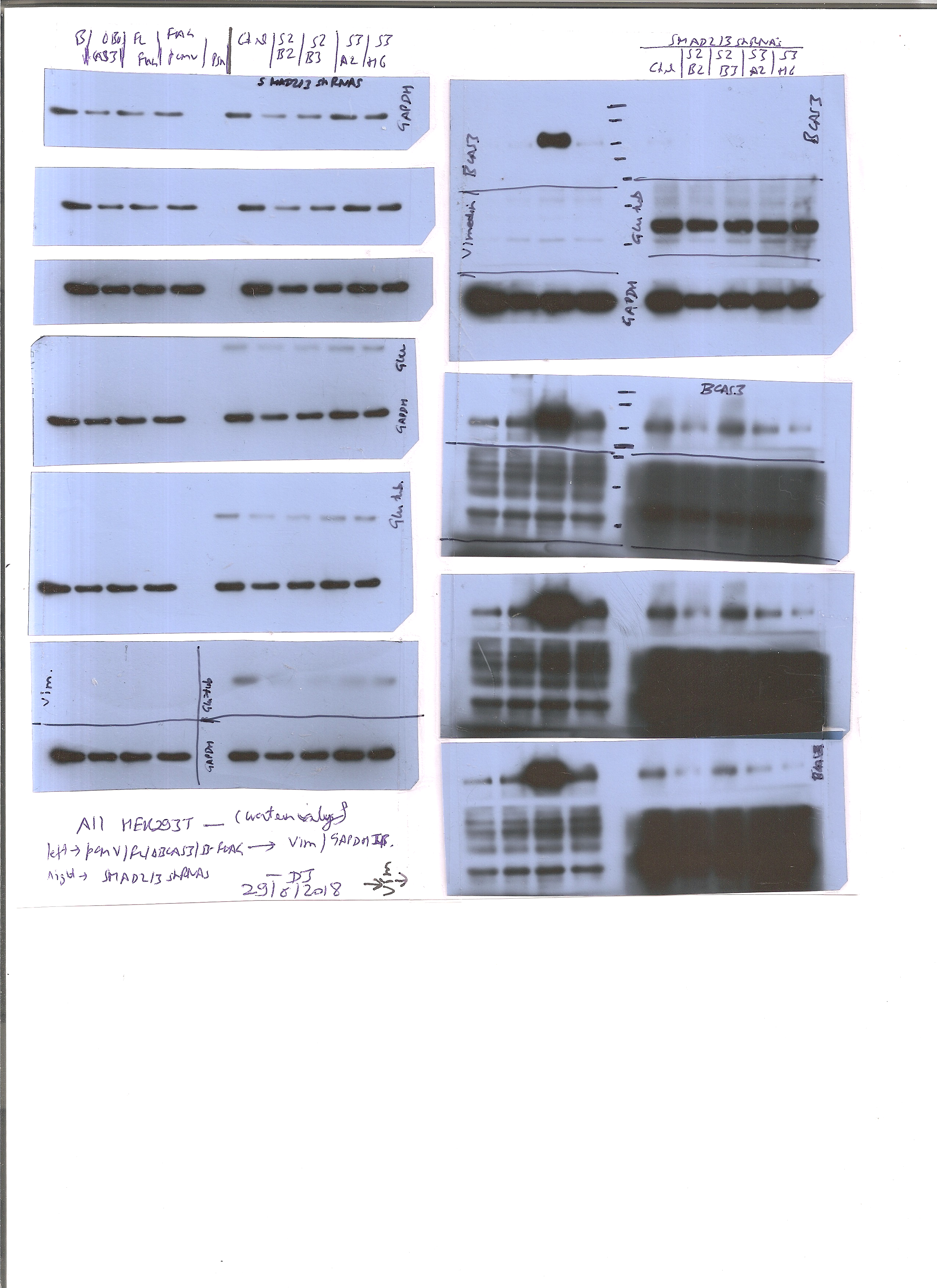

Supplement: Figure 4—source data 2. [file elife-98257-fig4-data2.zip › Fig4_SourceData1_raw/Fig4D_Rudhira_raw.tiff]

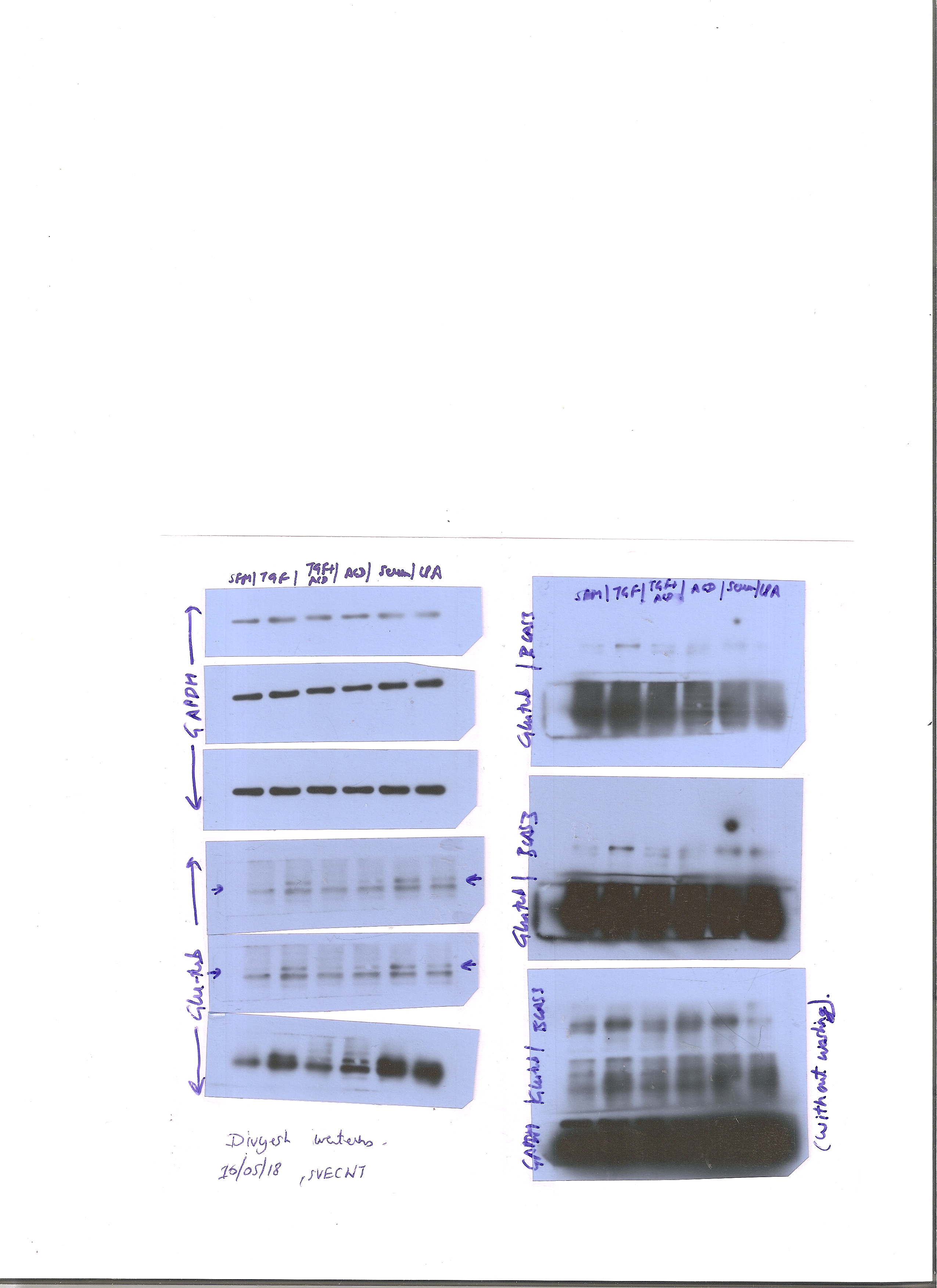

Supplement: Figure 4—source data 2. [file elife-98257-fig4-data2.zip › Fig4_SourceData1_raw/Fig4B_raw.tiff]

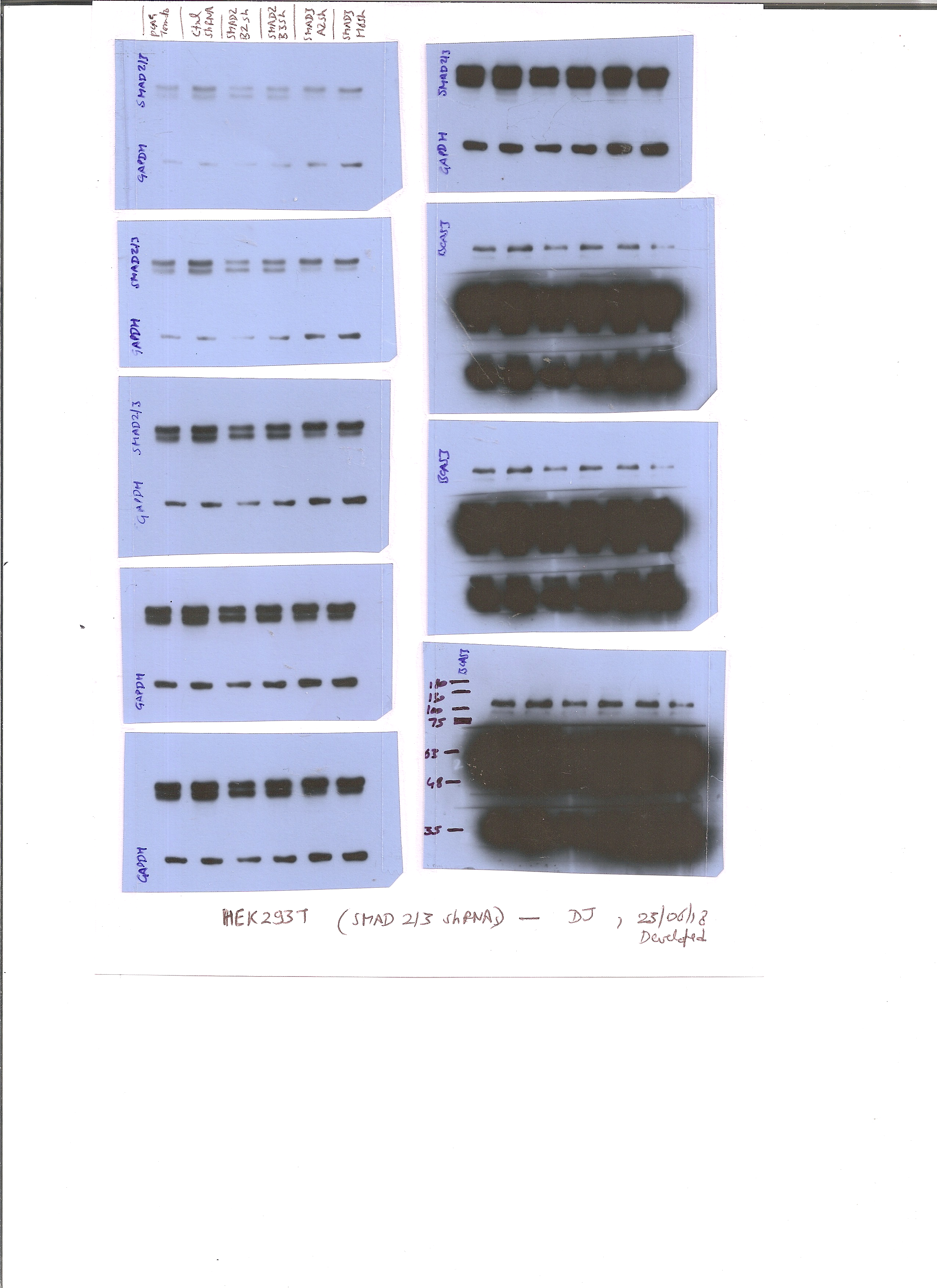

Supplement: Figure 4—source data 2. [file elife-98257-fig4-data2.zip › Fig4_SourceData1_raw/Fig4D_Smad23 and GAPDH_raw.tiff]

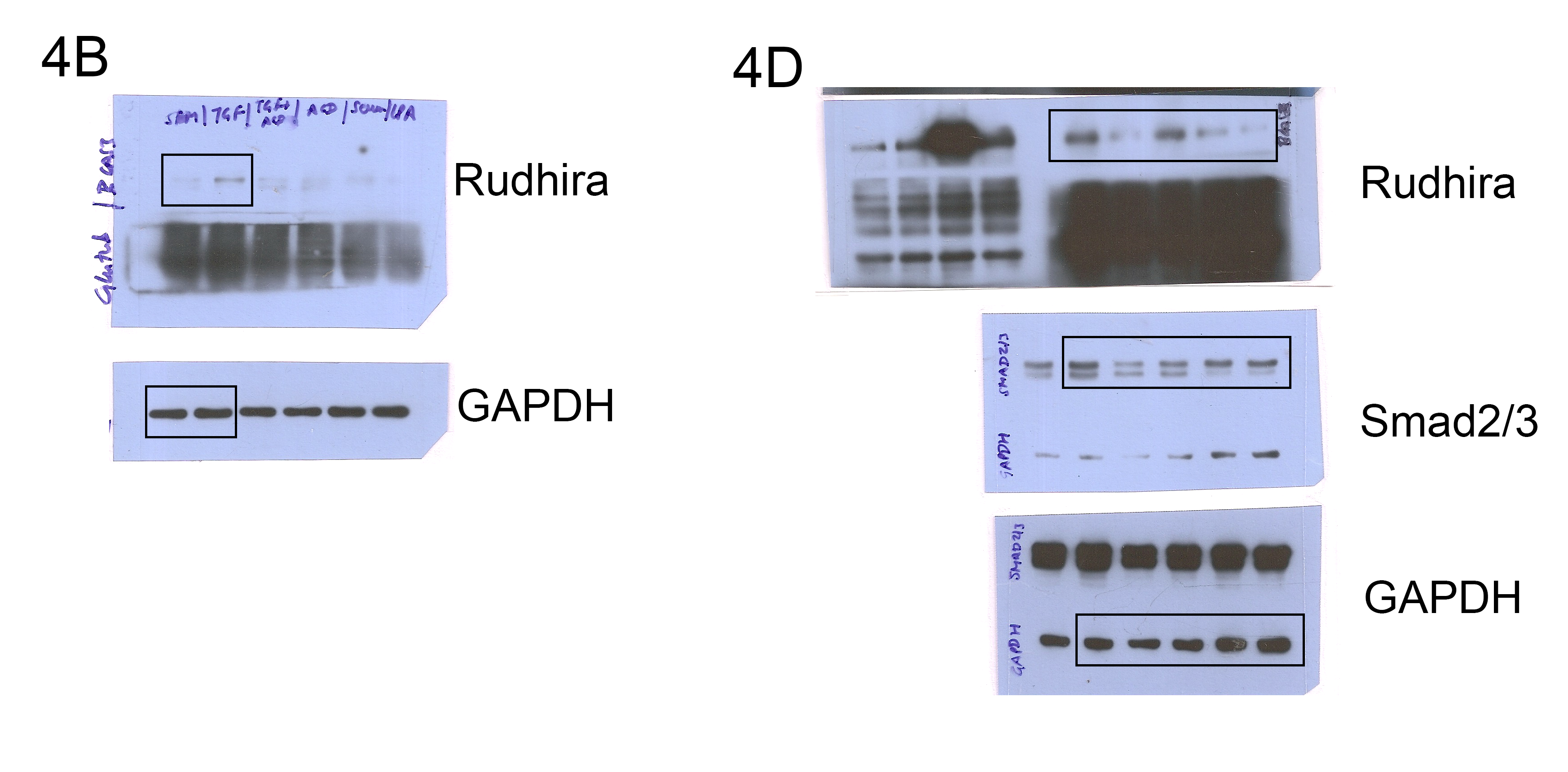

Supplement: Figure 4—source data 3. [file elife-98257-fig4-data3.zip › Fig4_SourceData2/Fig4_SourceData2.tif]

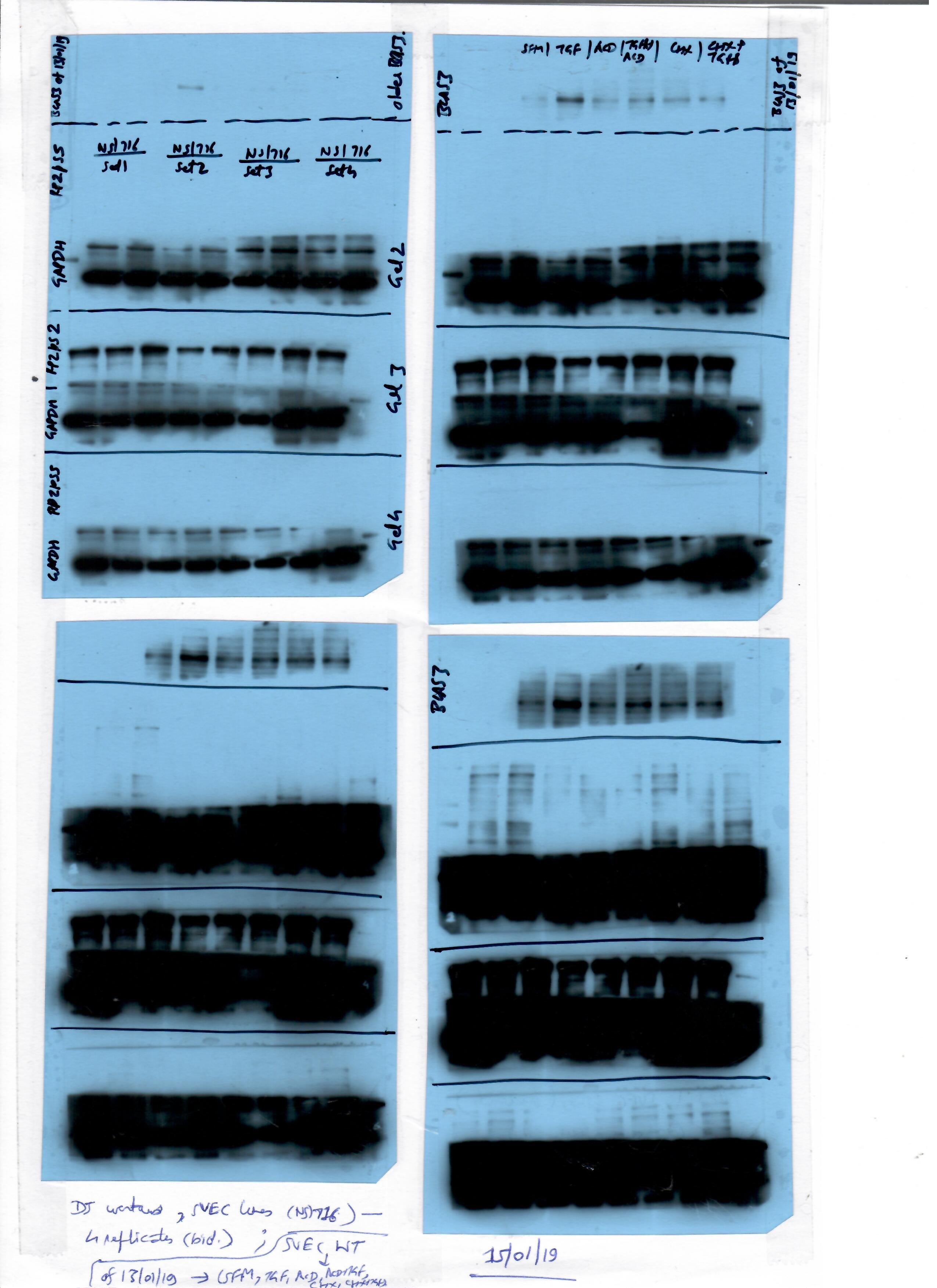

Supplement: Figure 5—source data 2. [file elife-98257-fig5-data2.zip › Fig5 and Fig4-Fig Supple1_SourceData1_raw/Fig5A and Fig4-Fig Supple 1C_Rudhira_raw.tiff]

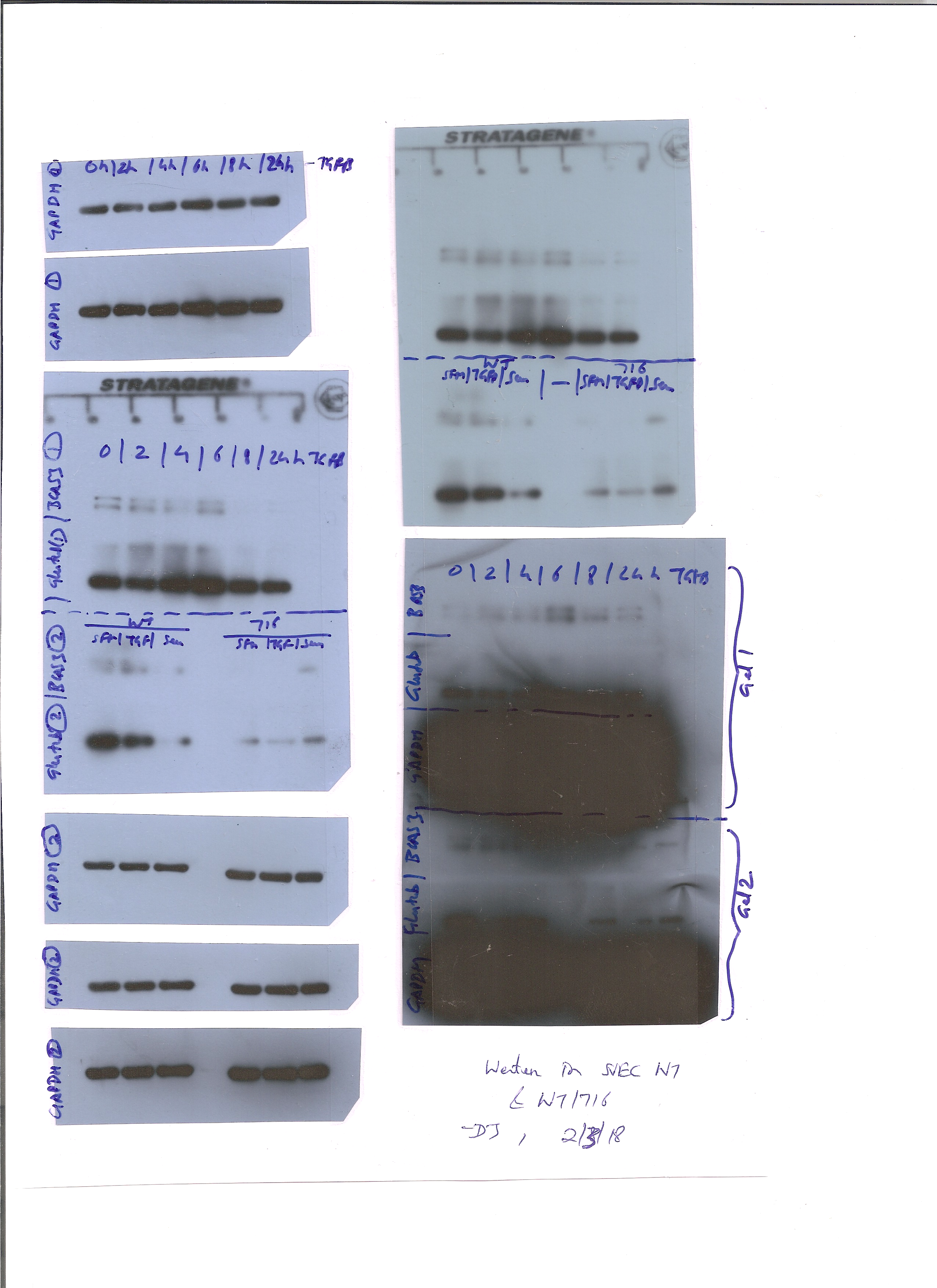

Supplement: Figure 5—source data 2. [file elife-98257-fig5-data2.zip › Fig5 and Fig4-Fig Supple1_SourceData1_raw/Fig5C and Fig4-Fig Supple 1D_raw.tiff]

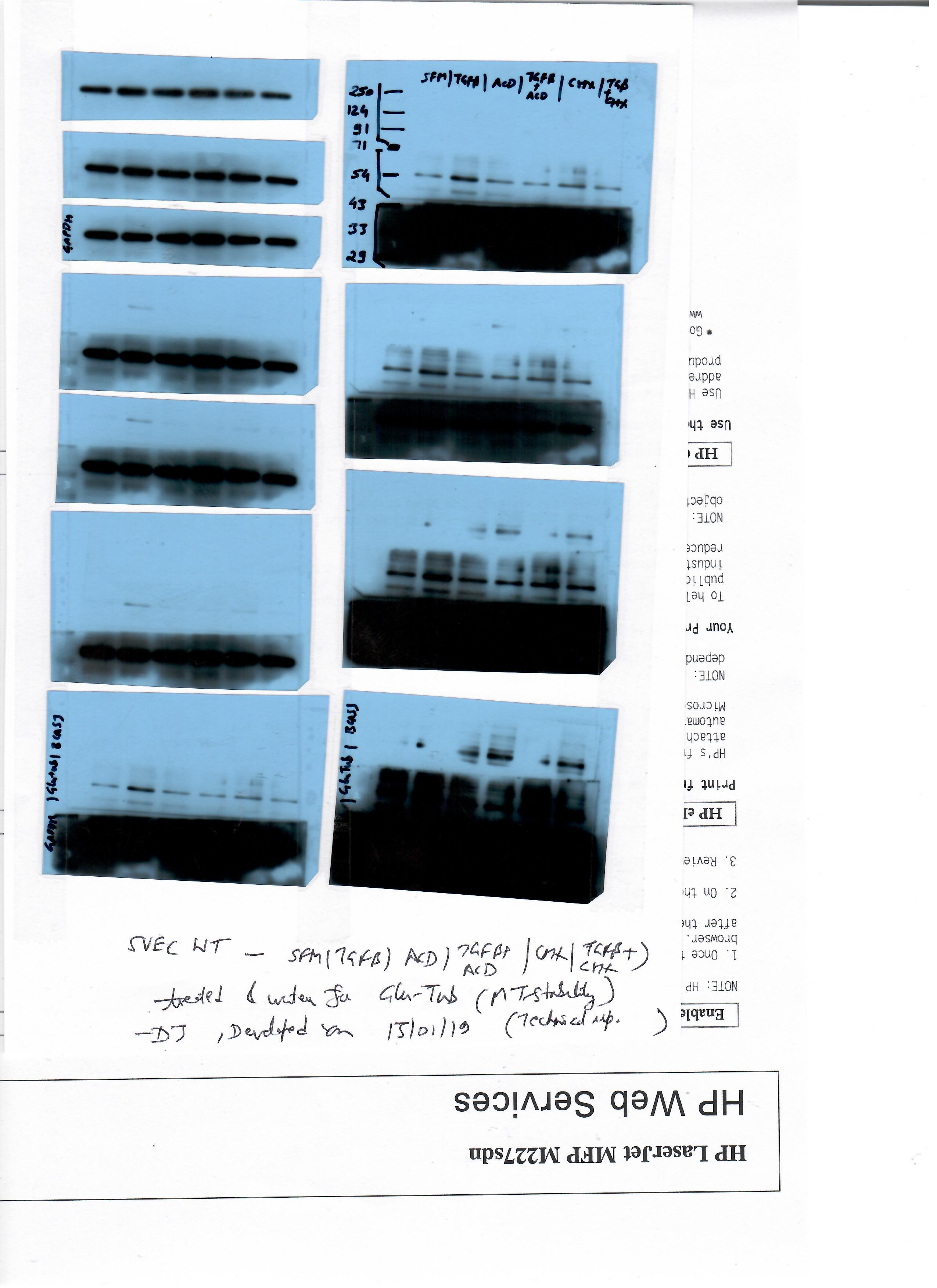

Supplement: Figure 5—source data 2. [file elife-98257-fig5-data2.zip › Fig5 and Fig4-Fig Supple1_SourceData1_raw/Fig5A and Fig4-Fig Supple 1C_Glu-Tub, GAPDH_raw.tiff]

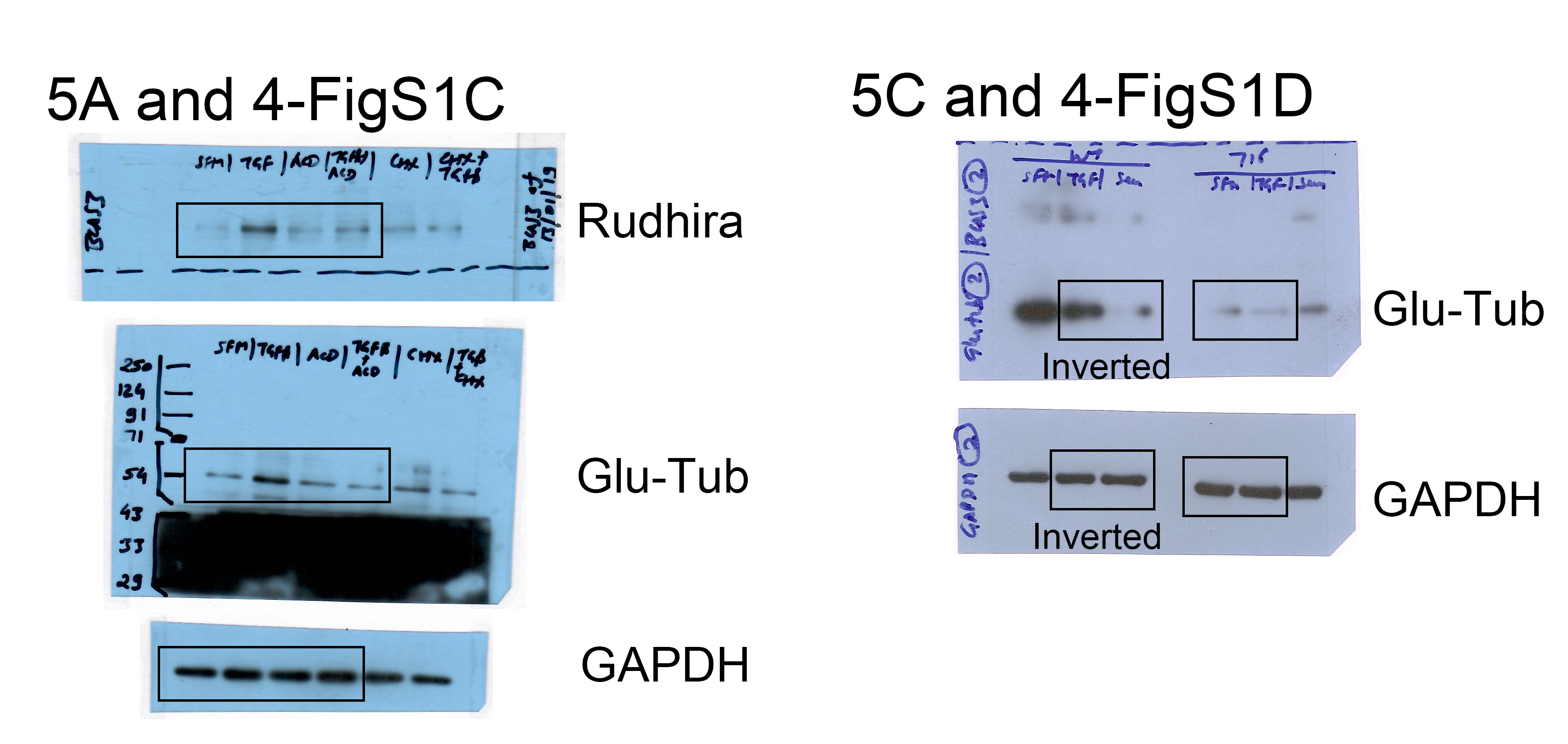

Supplement: Figure 5—source data 3. [file elife-98257-fig5-data3.zip › Fig5 and Fig4-Fig Supple1_SourceData2/Fig5 and Fig4-Fig Supple1_SourceData2.tif]
