# Supplementary material for: Rudhira-mediated microtubule stability controls TGFβ signaling during mouse vascular development
Source: eLife. 2025 May 15;13:RP98257. doi: 10.7554/eLife.98257 (PMC12080998; doi:10.7554/eLife.98257)
Supplement: Supplementary file 1. — (a) The oligonucleotide sequence of the Smad2 and Smad3 shRNAs. (b) Primers used for quantitative RT-PCR (qRT-PCR) analysis. (c) Predicted transcription factor binding sites in mouse and human rudhira/BCAS3 promoters, obtained from JASPAR bioinformatics tool. [file elife-98257-supp1.docx]

**Rudhira-mediated microtubule stability controls TGFβ signaling during mouse vascular development.**

Divyesh Joshi^1#^, Preeti Jindal^1#^, Ronak Shetty^1^ and Maneesha S. Inamdar^1,2*^

^1^Jawaharlal Nehru Centre for Advanced Scientific Research, Bangalore, India;^2^Institute for Stem Cell Science and Regenerative Medicine (inStem), Bangalore, India.

**Supplementary Files**

**Supplementary File 1a. The oligonucleotide sequence of the *Smad2* and *Smad3* shRNAs.**

| **Gene target** | **Oligonucleotide sequence in the shRNA (target sequence in red)** |
| --- | --- |
| *Smad2 sh#1* | CCGGCGATTAGATGAGCTTGAGAAACTCGAGTTTCTCAAGCTCATCTAATCGTTTTTG |
| *Smad2 sh#2* | CCGGCCTAAGTGATAGTGCAATCTTCTCGAGAAGATTGCACTATCACTTAGGTTTTTG |
| *Smad3 sh#1* | CCGGGCCTCAGTGACAGCGCTATTTCTCGAGAAATAGCGCTGTCACTGAGGCTTTTTG |
| *Smad3 sh#2* | CCGGGAGCCTGGTCAAGAAACTCAACTCGAGTTGAGTTTCTTGACCAGGCTCTTTTTG |

**Supplementary File 1b. Primers used for qRT-PCR analysis.**

| **Gene** | **Forward primer (5’ to 3’)** | **Reverse primer (5’ to 3’)** |
| --- | --- | --- |
| *rudhira* | TCCTACATGGAGAGCGTCG | GGAGGCTCATTTCCAGTGC |
| *smurf2* | AGACTGGTGTGAGCACATGG | CTTCCTGTTGCGGTATTGCG |
| *smad6* | GGGTGAATTCTCAGACGCC | GGTCGTACACCGCATAGAGG |
| *smad7* | CGGAAGTCAAGAGGCTGTGT | GACAGCCTGCAGTTGGTTTG |
| *pai1* | CTGTGCCCATGATGGCTCAG | ATGAACATGCTGAGGGTGTC |
| *mmp9* | GTCCAGACCAAGGGTACAGC | ATACAGCGGGTACATGAGCG |
| *gapdh* | GGTGAGGCCGGTGCTGAG | TGATGTCATCATACTTGGCAGG |

**Supplementary File 1c. Predicted Transcription factor binding sites in mouse and human**

***rudhira/BCAS3* promoters, obtained from JASPAR bioinformatics tool.**

| **Name** | **Position (from TSS)** | **Score** | **Background score** | **p-value** | **Matrix ID (JASPAR)** | **Strand** | **Predicted sequence** |
| --- | --- | --- | --- | --- | --- | --- | --- |
| MAX::MYC | -428 | 0.959466 | 0.823 | 0.00252271 | MA0059.1 | - | AAGCACGTGGA |
| MYC | -429 | 0.959143 | 0.827 | 0.00590903 | MA0147.3 | + | GTCCACGTGCTT |
| TWIST1 | -651 | 0.988341 | 0.854 | 0.00194672 | MA1123.2 | + | TTTCCAGATGTTC |
| HIF1A | -427 | 0.952744 | 0.849 | 0.0315803 | MA1106.1 | + | CCACGTGCTT |
| FOXP1 | -386 | 0.992493 | 0.876 | 0.0124319 | MA0481.3 | + | ATGTAAACACA |
| FOXF2 | -515 | 0.932307 | 0.826 | 0.0101525 | MA0030.1 | + | GGAAGGTAAACACA |
| MAX::MYC | -431 | 0.965445 | 0.823 | 0.00084454 | MA0059.1 | - | GAGCACGTGGA |
| MYC | -432 | 0.97587 | 0.831 | 0.00370719 | MA0147.3 | + | GTCCACGTGCTC |
| TWIST1 | -412 | 0.88988 | 0.849 | 0.19157 | MA1123.2 | + | GAACCAGCTGTCG |
| HIF1A | -430 | 0.963428 | 0.856 | 0.0274206 | MA1106.1 | + | CCACGTGCTC |
| FOXP1 | -389 | 0.989613 | 0.864 | 0.0168508 | MA0481.3 | + | ATGTAAACAGA |
| FOXF2 | -518 | 0.932307 | 0.817 | 0.0116588 | MA0030.1 | + | GGAAGGTAAACACA |
